# Modality-Attention Promotes the Neural Effects of Precise Timing Prediction in Early Sensory Processing

**DOI:** 10.3390/brainsci13040610

**Published:** 2023-04-03

**Authors:** Jiayuan Meng, Xiaoyu Li, Yingru Zhao, Rong Li, Minpeng Xu, Dong Ming

**Affiliations:** 1 Academy of Medical Engineering and Translational Medicine, Tianjin University, Tianjin 300072, China; 2 College of Precision Instruments and Optoelectronics Engineering, Tianjin University, Tianjin 300072, China

**Keywords:** timing prediction (TP), modality attention (MA), event-related potential (ERP), inter-trial coherence (ITC), event-related spectral perturbation (ERSP)

## Abstract

Precise timing prediction (TP) enables the brain to accurately predict the occurrence of upcoming events in millisecond timescale, which is fundamental for adaptive behaviors. The neural effect of the TP within a single sensory modality has been widely studied. However, less is known about how precise TP works when the brain is concurrently faced with multimodality sensory inputs. Modality attention (MA) is a crucial cognitive function for dealing with the overwhelming information induced by multimodality sensory inputs. Therefore, it is necessary to investigate whether and how the MA influences the neural effects of the precise TP. This study designed a visual–auditory temporal discrimination task, in which the MA was allocated to visual or auditory modality, and the TP was manipulated into no timing prediction (NTP), matched timing prediction (MTP), and violated timing prediction (VTP) conditions. Behavioral and electroencephalogram (EEG) data were recorded from 27 subjects, event-related potentials (ERP), time–frequency distributions of inter-trial coherence (ITC), and event-related spectral perturbation (ERSP) were analyzed. In the visual modality, precise TP led to N1 amplitude and 200–400 ms theta ITC variations. Such variations only emerged when the MA was attended. In auditory modality, the MTP had the largest P2 amplitude and delta ITC than other TP conditions when the MA was attended, whereas the distinctions disappeared when the MA was unattended. The results suggest that the MA promoted the neural effects of the precise TP in early sensory processing, which provides more neural evidence for better understanding the interactions between the TP and MA.

## 1. Introduction

Sub-second timing prediction (TP) enables humans to accurately predict the occurrence of upcoming events. It can speed up behaviors, facilitate perceptions, and optimize the allocations of cognitive resources effectively [1,2]. The cerebellum and basal ganglia are major neural structures responsible for timing prediction, which plays a key role in single-interval and rhythmic timing, respectively [3,4]. Supplementary motor areas and the medial entorhinal cortex also contribute to the TP process [5,6,7,8]. Neural responses of precise TP within a single sensory modality have been widely studied [1,2]. However, less is known about how the TP works when the brain is concurrently faced with multimodality sensory inputs. Modality attention (MA) is the brain’s ability to prioritize information from a specific sensory modality, which can mitigate computational burdens induced by multimodality sensory inputs [9]. Therefore, it is of vital importance to investigate whether and how the MA influences the neural effects of the precise TP.

Precise TP modulates both the pre-stimulus and evoked neural responses. Event-related potential (ERP) studies reported that the contingent negative variation (CNV) could index subjects’ time estimation ability [10,11,12]. Neural oscillation studies highlighted that low frequency activities (<15 Hz) can represent TP. Specifically, the delta (1–3 Hz) phase reset had stronger inter-trial coherence (ITC) at the predicted moment during both rhythmic and non-rhythmic tasks [13,14]. Frontal theta (4–7 Hz) ITC was modulated by the prediction error magnitude when subjects undertook a visual temporal learning task, suggesting a close association with updating temporal information [15]. The alpha (8–15 Hz) phase immediately before the visual stimulation was guided by top-down timing prediction [16,17], and alpha power changes were also found in some timing prediction studies [18,19]. Inspired by the predictive coding theory, which regards the brain as a prediction machine that can actively infer the external world and attempt to match incoming sensory inputs with top-down predictions [20,21,22], there is growing agreement that the TP is a neural implementation of the predictive coding in the time domain [23,24]. Thus, comparing the evoked responses, which were induced by the stimuli emerging just at (matched timing prediction, MTP) and not at (violated timing prediction, VTP) the predicted moment, is promising to better reflect the neural effect of the precise TP. This hypothesis was supported by the observation that N1-P2 amplitudes indexed subjective time more accurately than the CNV [25]. In our previous study, the TP was manipulated into different conditions by a visual task. In the early sensory processing stage (less than 400 ms after the target onset), the MTP conditions resulted in similar ERP profiles with the no timing prediction (NTP) conditions, whereas VTP condition suppressed N1 and enhanced N2 in the occipital brain area [26]. However, this TP neural effect was observed when there were only visual stimuli. It remains unclear whether such an opposing effect still occur when the brain is concurrently faced with audio-visual stimuli, and whether neural responses in auditory modality are similar to that of visual or not.

MA is a crucial cognitive function for dealing with the overwhelming information induced by multimodality sensory inputs. When both the auditory and visual stimulations were presented, it is possible that MA would optimize sensory processing in a specific modality. However, previous studies concentrated more on how the MA influenced the multisensory integration of time information [27,28]. Neural evidence is still lacking regarding if and how MA influences the processing of the precise TP, especially in two aspects. First, it remains controversial whether the precise TP neural effect is independent of the MA, or if it performs differently under attended and unattended conditions. For this, an EEG study concurrently manipulated the visual–tactile attention and rhythmic-based timing prediction within an experiment. TP began to work preceding the MA, and the two processes had opposing effects in modulating early evoked responses [29]. However, to the best of our knowledge, there have not been any studies investigating how auditory–visual attention modulates the neural responses of single-interval precise TP. Second, previous studies have suggested that the precise TP led to changes in either early evoked ERPs or low-frequency neural oscillations. However, it remains unclear which features may better reflect the neural effects of TP. Investigation is needed to determine how these features change when modality attention is attended or unattended.

This study investigated how audio–visual modality attention influences the neural effects of the single-interval precise TP. EEGs from 27 subjects were recorded and analyzed; ERPs, time–frequency analyses of ITC, and event-related spectral perturbation (ERSP) [30] were calculated and compared, respectively. This experiment included three TP conditions: NTP, MTP, and VTP. MA conditions included visual-attended (Va), visual-unattended (Vua), auditory-attended (Aa), auditory-unattended (Aua). We found (i) in the visual modality, the TP led to the opposing N1-N2 performance only when the MA was attended. (ii) In the auditory modality, when the MA was attended, the MTP had the largest neural responses in P2 temporal window among distinct TP conditions, and these distinctions disappeared when the MA was unattended. (iii) Low-frequency ITC could better reflect the modulations of both the TP and MA. These results suggest that the MA can promote the neural effects of precise single-interval TP in early sensory processing.

## 2. Materials and Methods

### 2.1. Participants

Twenty-seven right-handed students from Tianjin University, aged between 18 and 26 years, were recruited for the experiment. Participants had to have normal or corrected-to-normal vision and be free from psychological or neurological diseases. Experimental procedures were approved by the Institutional Review Board at Tianjin University. All possible consequences were explained, and the written informed consent was obtained from all the participants.

### 2.2. Stimuli

**Figure 1:**
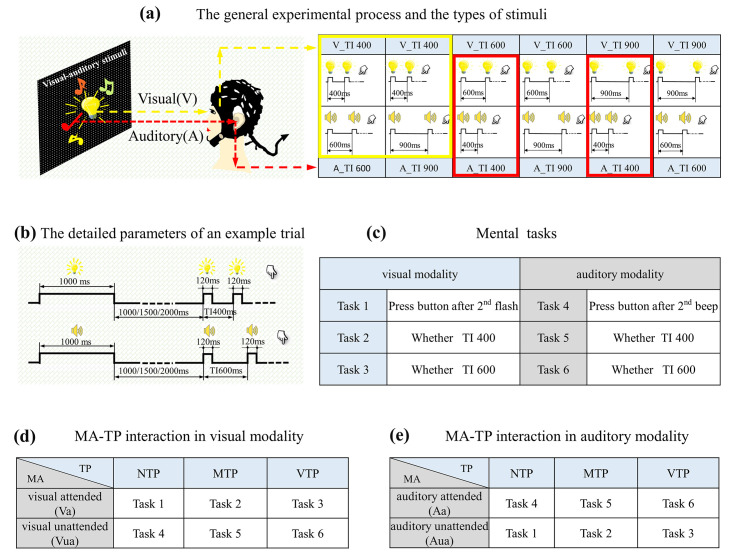
Illustration of the experimental design: (**a**) The general experimental process: There were six kinds of visual–auditory combinations in total, which are listed in the table. The yellow and red boxes indicate the trials selected for visual and auditory analyses, respectively. (**b**) An example of the detailed parameters. (**c**) The list of mental tasks, with 1–6 used to represent the tasks described above. These numbers did not represent the presentation order of the tasks. (**d**) The manipulation of modality attention (MA) and precise timing prediction (TP) in visual, and (**e**) auditory modality.

This study designed a visual–auditory temporal discrimination task. The stimuli included flashing LED and buzzer, driven by a chronometric FPGA platform (Cyclone II: EP2C8T144C8), with a time precision of 20 ns in controlling. Visual stimuli were presented by a 15 × 15 mm^2^ LED placed at the eye level, 80 cm away from participants. Auditory stimuli were generated by a buzzer located at the same position as LED. As Figure 1a shows, there were three time intervals (TI) between first and second stimuli, (400 ms, 600 ms, and 900 ms). The first visual and auditory stimuli were presented concurrently, whereas the second were not. The visual TI differed from auditory TI, which formed six flash–buzzer combinations. An example of a single trial was shown in Figure 1b. In this example, the trial started with a visual–auditory cue lasting for 1000 ms, then there was a blank period with a random duration selected from 1000 ms, 1500 ms, or 2000 ms; the random blank made the onset of first stimulus unpredictable. Next, the first visual–auditory stimuli emerged concurrently, and then the second visual and auditory stimuli appeared, but with different TIs. Finally, there was a pseudo-random period between 1600 and 3000 ms before the next trial. A block consisted of 30 trials. Each visual-auditory combination emerged for five trials, and all 30 trials were presented randomly.

### 2.3. Experimental Procedure

The formal experiment took place in an electrically shielded room and included six mental tasks. As described in Figure 1c (left), the first three were visual tasks. For the first task, participants were required to indicate the onset of the second flash and if so, no specific moment was predicted. For the second task, participants had to indicate whether the second flash appeared 400 ms after first. Under this condition, 400 ms after first flash was the only predicted moment. For the third task, participants had to indicate whether the second flash appeared 600 ms after first flash, for example 600 ms after first flash was the predicted moment. Another three tasks were auditory tasks. For the first auditory task, participants were required to indicate the onset of second beep. For the second auditory task, participants had to indicate whether the second beep appeared 400 ms after first. For the third auditory task, participants had to indicate whether second beep appeared 600 ms after first flash. During auditory discrimination tasks, participants were required to maintain their sight on the visual stimuli at all the time, so that their visual inputs were completely identical to those in visual tasks. Participants made their decisions by pressing buttons with right/left thumb, which was balanced across blocks. Each task had four blocks. There were twenty-four blocks in total, all the twenty-four blocks were conducted randomly.

A precise-enough predictive template is a prerequisite for successfully manipulating the precise TP. For this reason, participants were trained for three days before the formal experiment; only when the discrimination accuracy was more than 80% could they start the formal experiment. On the first training day, participants first learnt about the three timing intervals and tried to discriminate them (i.e., TI400, TI600, and TI900), by watching the double-flash with a single timing interval, and specific TI was cued by the experimenter before each trial. After ~20 min of learning, they were then asked to determine which TI the presented double flash was by pressing buttons. On the second training day, subjects participated in a visual or auditory temporal discrimination task, in which they were required to judge whether the actual double-flash/beep was TI400 or TI600. Notably, in each block, there were 10–15 trials, the timing of which was randomly selected from 300 ms, 500 ms, and 800 ms. The aim of adding these untrained TIs into the training block is to avoid the participants from realizing there were only three kinds of actual stimuli in the formal test and allocated more attentional resources to the three specific moments. On the third training day, subjects underwent the same training as the second day.

### 2.4. Experimental Design for Forming Distinct MA-TP Conditions

MA was manipulated by the task-relevance. In visual tasks, attentional resources were allocated to visual modality. For visual tasks 1–3, they were visual attended (Va) (Figure 1d upper), but auditory unattended (Aua) (Figure 1e lower). In auditory tasks, attention was allocated to auditory modality. For tasks 4–6, they were auditory attended (Aa) (Figure 1d lower), but visual unattended (Vua) (Figure 1e upper).

Distinct TP conditions were formed by the interactions between mental tasks and actual onset moment of the second stimulus. Trials containing 400 ms TI were extracted, as the yellow (for visual modality) and red (for auditory modality) boxes in Figure 1a shows. This means that the actual stimuli for analyzing were identical; but the predicted moment in subjects’ minds varied with mental tasks. Specifically, in tasks 1 and 4, there was no specific oriented moment, i.e., NTP condition. In this condition, induced neural activities were the least influenced by top-down process, which can be a baseline for studying how the TP changes evoked neural responses. In tasks 2 and 5, the only predicted moment was 400 ms after first stimulation, so the actual stimulus emerged exactly at the predicted moment, i.e., MTP condition. In tasks 3 and 6, 600 ms after first stimulation was the only predicted moment, which means that the actual stimulus occurred before the predicted moment, i.e., VTP condition.

In summary, the tasks for forming distinct MA-TP conditions were listed in Figure 1d,e. There were 12 MA-TP conditions in total: Va-NTP, Va-MTP, Va-VTP, Vua-NTP, Vua-MTP, Vua-VTP, Aa-NTP, Aa-MTP, Aa-VTP, Aua-NTP, Aua-MTP, Aua-VTP.

### 2.5. EEG Recording and Pre-Processing

EEG was recorded using a 64-electrode Neurocan Synamps2 system at a sample rate of 10,000 Hz and was notch-filtered at 50 Hz. All electrodes were positioned on the scalp according to the International 10–20 system, and were all referenced to the tip of nose and grounded to the frontal brain area. Additional bipolar electrodes registered the electro-oculogram (EOG). An independent component analysis (ICA) was used to reject eye movement artifacts. Eye-related components were identified by comparing individual ICA components with EOG channels and by visual inspection. To collect qualified EEG signals, the impedance levels of all the electrodes were less than 10 kΩ.

In pre-processing, EEG data were filtered by a FIR Ⅰ low-pass filter cutting at 40 Hz and down-sampled to 200 Hz. According to the experimental design, the TP mainly worked after the second onset of TI400 double-stimulus, whereas responses to the first stimulus was almost not influenced. Therefore, the second stimulus onset was defined as the zero point. The correct trials with a reaction time less than 80 ms (relative to the zero-time point) were defined as the qualified trials; each MA-TP condition contained 35–40 qualified trials for subsequent EEG analyses in total.

### 2.6. Data Processing and Analyses

This study analyzed EEG from O1, OZ, and O2 electrodes for probing the evoked responses in visual modality, and EEG from F1, FZ, and F2 electrodes for auditory responses. Choosing these electrodes was based on earlier studies that investigated visual or auditory neural responses using these electrodes [26,31,32].

For ERP analyses, baseline correction was performed using a 100 ms pre-stimulus baseline for the ERPs induced by the first and second stimulus, respectively. Such baseline correction was because this study mainly investigated the evoked responses rather than the CNVs.

The ERP technique, time-frequency analyses were used to measure the evoked neural responses under distinct MA-TP conditions. In visual modality, N1 component induced by first and second flash, and N2 component induced by second flash, were selected for further analyses. According to the separations of visual ERP profiles, the temporal windows for first N1, second N1, second N2 were defined as 140–200 ms after the first flash (i.e., −240 to −200 ms relative to zero point), 120–190 ms and 200–300 ms after second flash, respectively. In auditory modality, the temporal windows for P2 component induced by the first and second beep were defined as −230 to −180 ms and 110–250 ms, respectively. The ERP amplitude was calculated as the mean amplitude within specific temporal window.

The ITC and ERSP were calculated to show the event-related neural dynamics with a time-frequency distribution. ITC measures the phase synchronization to a set of experimental events to which EEG trials are time-locked, and it values between 0 and 1. The larger an ITC value is, the stronger the phase synchronization is. The ITC can be calculated as Equation (1). Moreover, the ERSP was used to visualize event-related changes in spectral power over time, with a baseline covering 100 ms before the first stimulus. It was calculated as Equation (2)

(1) $$\mathrm{ITC}\left(f,t\right)=\frac{1}{N}\left|\sum_{i=1}^{k}\frac{F_{i}\left(f,t\right)}{\left|F_{i}\left(f,t\right)\right|}\right|\;$$

(2) $$\mathrm{ERSP}\left(f,t\right)=\frac{1}{N}\sum_{i=1}^{k}{\left|F_{i}\left(f,t\right)\right|}^{2}\;$$

According to the inspection of the time-frequency distribution, in visual modality, three temporal windows were selected for ITC analyses (−300 to −150 ms, 100–200 ms and 200–300 ms relative to zero point, respectively). In auditory modality, three temporal windows were selected for ITC analyses (−300 to −150 ms, 100–200 ms and 200–400 ms, respectively). In ERSP analysis, the temporal windows of −100 to −200 ms, 100–200 ms and 200–400 ms were selected for both the visual and auditory analyses. As to the frequency information, 1–3 Hz, 4–8 Hz, 8–14 Hz and 15–30 Hz were defined as frequency windows of delta, theta, alpha and beta band, respectively.

### 2.7. Statistical Analyses

In behavioral analysis, the paired-samples T test was used to make comparisons between visual and auditory tasks; one-way repeated-measures analysis of variance (ANOVA) was used for comparing reaction time and accuracy rate in NTP, MTP, and VTP conditions. EEG were analyzed by two-way repeated ANOVA. In visual modality, there were four separate hypotheses: there should be an MA-TP interaction on the visual (i) N1 amplitude, (ii) N2 amplitude, (iii) ITC, and (iv) ERSP. Similarly, there were three separate hypotheses: there should be an MA-TP interaction on the auditory (i) P2 amplitude, (ii) ITC, and (iii) ERSP. For each hypothesis, if the interactive effect did not exist, the main effect of modality attention and timing prediction would be tested, respectively. If the interactive effect existed, we then tested the simple effect of timing prediction under attended and unattended conditions, respectively. For each hypothesis, the Bonferroni method was used for multiple comparison test.

## 3. Results

### 3.1. Behavioral Results

**Figure 2:**
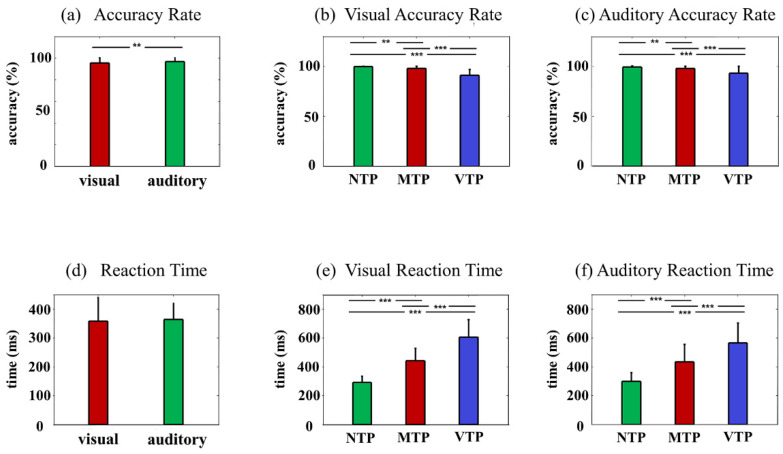
Behavioral results: (**a**) Comparison between reaction times of visual and auditory tasks; (**b**) In the visual and (**c**) auditory modality, the comparisons of reaction time under distinct TP conditions; (**d**) Accuracy comparison between the visual and auditory tasks; and (**e**) In the visual and (**f**) auditory modality, accuracy comparisons under distinct TP conditions. Statistical significance: ** 0.01 < *p* < 0.1; *** *p* < 0.001.

A total of 27 adults were included in the experiment, 14 of which were female. Reaction time and accuracy rate were analyzed to examine (i) whether the visual and auditory tasks led to similar behavioral results; (ii) whether TP manipulations were effective. Reaction time was defined as the period between button press and second stimulus onset. Accuracy rate was the ratio of correct trials with a reaction time less than 80 ms to the total trials. As Figure 2a shows, accuracy rates were high for both visual and auditory tasks (95.69% and 96.73%, respectively). Reaction times were 358.15 ms and 364.53 ms for visual and auditory tasks, respectively.

The TP was manipulated via the interactions between the predicted and actual onset moment. This means that if the manipulation was successful, subjects would behave differently even when faced with the identical stimuli. This study mainly investigated behavioral results induced by the TI400 actual stimuli, as other longer time intervals would face the problem of information disclosure and would not fully reflect the TP effects. As Figure 2b,e shows, in Va conditions, the reaction times were 291.95 ms, 442.28 ms and 606.58 ms, for NTP, MTP and VTP, respectively; accuracy rates were 99.9%, 98.2% and 91.2%. In Aa conditions, the reaction times were 300.40 ms, 435.48 ms and 566.44 ms for NTP, MTP, VTP, respectively; accuracy rates were 99.96%, 97.83% and 93.83. The NTP, which only had one choice rather than two, was the easiest mental task of all. Therefore, it is not surprising that the NTP had the shortest reaction times (Va: *F*(2,52) = 176.49, *p* < 0.001, NTP vs. MTP/VTP: *p* < 0.001, Aa: *F*(2,52) = 91.54, *p* < 0.001; NTP vs. MTP/VTP: *p* < 0.001, all after the Bonferroni correction), and the highest accuracy rates (Va: *F*(2,52) = 50.147, *p* < 0.001; NTP vs. MTP: *p* = 0.003; NTP vs. VTP: *p* < 0.001, Aa: *F*(2,52) = 16.353, *p* < 0.001; NTP vs. MTP: *p* = 0.007; NTP vs. VTP: *p* < 0.001, all after Bonferroni correction). Moreover, compared with VTP, the MTP condition had improved accuracy rates (visual: *p* < 0.001; auditory: *p* < 0.001) and reaction times (visual: *p* < 0.001; auditory: *p* < 0.001). This suggests that proper TP can improve behavioral performance. These results demonstrated that visual and auditory tasks had similar behavioral performances and confirmed the effectiveness of TP manipulations in both visual and auditory tasks.

### 3.2. ERP Analyses

**Figure 3:**
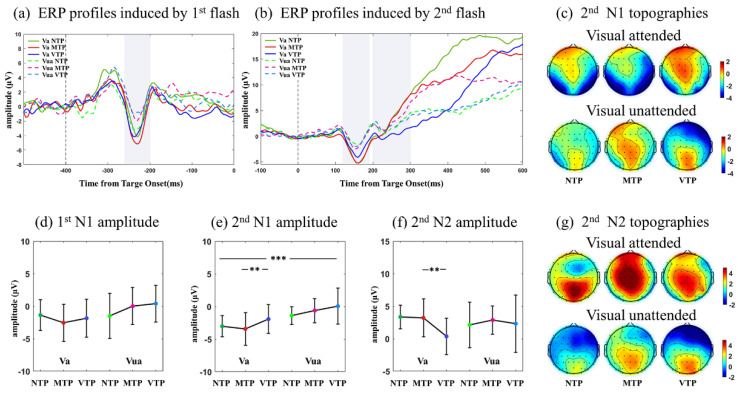
(**a**) Averaged ERPs of O1, OZ, O2 electrodes induced by the first flash. Va and Vua conditions were labeled by solid and dashed lines, respectively. Color green, red, blue represented NTP, MTP, VTP conditions, respectively. (**b**) ERPs of O1, OZ, O2 electrodes induced by the second flash. (**c**) N1 amplitude topographies induced by the second flash, the NTP, MTP, VTP conditions were placed in left, middle, right rows, respectively; the Va and Vua conditions were placed at upper and lower lines, respectively. (**d**) Comparisons of N1 amplitude induced by the first and (**e**) second flash. (**f**) Comparisons of N2 amplitude induced by the second flash. (**g**) N1 amplitude topographies induced by the second flash. Statistical significance: the asterisks at the top of each figure indicated the MA main effect; the asterisks above the Va condition (Va−NTP, Va−MTP, Va−NTP) indicated the TP simple effect under attended condition, when the MA-PT interactive effect existed. Statistical significance: ** 0.001 < *p* <0.01; *** *p* < 0.001.

ERPs and topographies in visual modality, included six MA-TP conditions (Va-NTP, Va-MTP, Va-VTP, Vua-NTP, Vua-MTP and Vua-VTP). We first analyzed the N1 amplitude in ERP profiles induced by the first flash (Figure 3a,d). No interactive effect, TP or MA main effect were found. Following the second flash, the N1 and N2 components revealed striking variations across conditions, their topographies revealed differences as well (Figure 3c,g). In the N1 period, obvious attentional enhancement (*F*(1,26) = 19.190, *p* < 0.001, *η*^2^ = 0.434) (Figure 3e) was observed. Moreover, in Va conditions, VTP led to much smaller N1 amplitude than the MTP (*F*(2,52) = 3.762, *p* = 0.035, MTP vs. VTP: *p* = 0.001, after Bonferroni correction), but no TP difference was found in Vua conditions. After N1, the attended NTP and MTP had an upward trend, whereas the attended VTP initially went down before rising again, which resulted in a more negative N2 component (*F*(2,52) = 4.087, *p* = 0.024, MTP vs. VTP: *p* = 0.001). However, in Vua condition, ERP profiles of the NTP, VTP and MTP were similar (Figure 3b).

**Figure 4:**
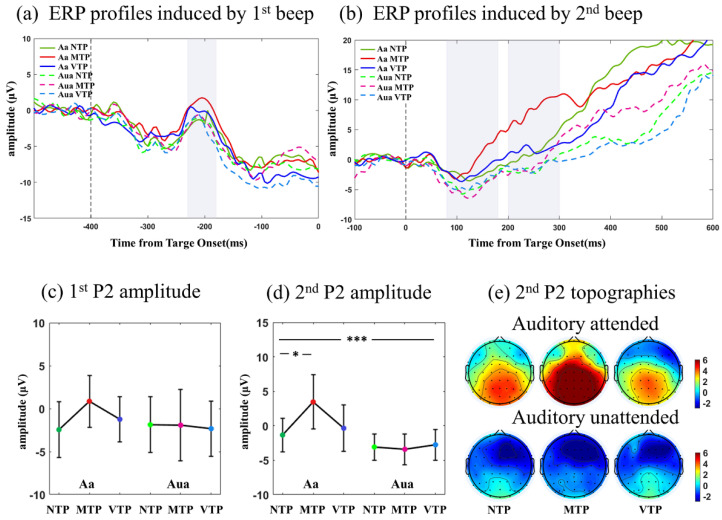
(**a**) Averaged ERPs of F1, FZ, F2 induced by the first beep. (**b**) Averaged ERPs induced by the second beep. (**c**,**d**) Comparisons of P2 amplitude induced by second beep. (**e**) Amplitude Topographies of P2 induced by second beep. Statistical significance: the asterisks at the top of each figure indicated the MA main effect; the asterisks above the Va condition (Va−NTP, Va−MTP, Va−NTP) indicated the PT simple effect under attended condition, when the MA−PT interactive effect existed. Statistical significance: * 0.01 < *p* < 0.05; *** *p* < 0.001.

ERPs and topographies in auditory modality can be seen in Figure 4 and included six MA-TP conditions: Aa−NTP, Aa−MTP, Aa−VTP, Aua−NTP, Aua−MTP, Aua−VTP. Auditory ERPs were mainly slow waves. By inspecting profiles, P2 component induced by the first and second beeps were selected for further analyses. Figure 4c shows there was no interactive effect, MA main effect or TP main effect after the first beep. Figure 4d was the comparisons of P2 induced by second beep. There was a significant interaction effect of the MA and TP (*F*(2,52) = 3.573, *p* = 0.035, $\eta^{2}$ = 0.125). In Aa condition the MTP response was much larger than that of NTP (*p* = 0.015 after Bonferroni correction), whereas the distinctions disappeared in Aua condition.

ERP analyses showed that in visual modality, there was an MA-TP interactive effect on the N1 and N2 amplitudes. The VTP resulted in smaller N1 and larger N2 only in Va condition. In auditory modality, there was an MA-TP interactive effect on P2 amplitude. The MTP led to a larger P2 component only in Aa condition.

### 3.3. ITC Time–Frequency Analyses

**Figure 5:**
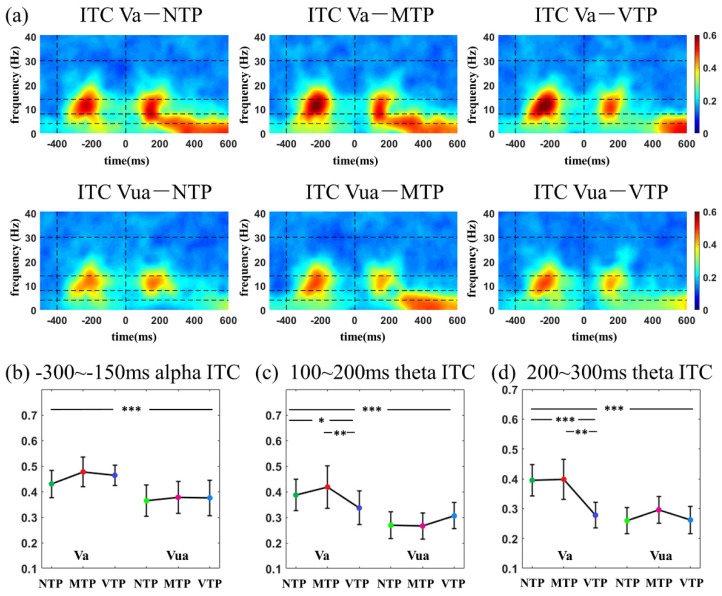
(**a**) ITC time−frequency distributions in visual modality; comparisons among distinct MA-TP conditions of (**b**)−300–−150 ms alpha ITC; (**c**) 100–200 ms theta ITC; (**d**) 200–300 ms theta ITC. Statistical significance: the asterisks at the top of each figure indicated the MA main effect; the asterisks above the Va condition (Va−NTP, Va−MTP, Va−NTP) indicated the TP simple effect under attended condition, when the MA−TP interactive effect existed. * 0.01 < *p* < 0.05; ** 0.001 < *p* < 0.01 *** *p* < 0.001.

Figure 5a shows visual ITC time-frequency distributions in six MA-TP conditions. For the ITCs induced by the first flash, there was no MA-TP interactive effect (theta: *F*(2,52) = 0.088, *p* = 0.916; alpha: *F*(2,52) = 1.595, *p* = 0.213). Only the attentional enhancement was found. Alpha ITC was larger in Va conditions than the Vua (*F*(1,26) = 21.09, *p* < 0.001, $\eta_{p}^{2}$ = 0.448). There was no TP main effect (theta: *F*(2,52)= 1.721, *p* = 0.189; alpha: *F*(2,52) = 1.875, *p* = 0.201). The first flash emerged randomly, resulting in an absence of precise TP. This suggests that the responses are not modulated by the TP. Distinct TP conditions revealed similar responses in-line with the experimental design.

ITCs induced by the second flash were then studied. There was a significant MA-TP interactive effect on the theta ITC in both the 100–200 ms (Figure 5c, *F*(2,52) = 8.027, *p* < 0.001, $\eta_{p}^{2}$ = 0.448) and 200–300 ms (Figure 5d, *F*(2,52) = 9.521, *p* < 0.001, $\eta_{p}^{2}$ = 0.276) temporal windows. In Va condition, the theta ITC of the VTP condition was smaller than others in both the 100–200 ms (*F*(2,52) = 10.195, *p* < 0.001; NTP vs. VTP: *p* = 0.033; MTP vs. VTP: *p* = 0.002, both after Bonferroni correction) and 200–300 ms (*F*(2,52) = 15.469, *p* < 0.001; NTP vs. VTP: *p* < 0.001; MTP vs. VTP: *p* = 0.001, both after the Bonferroni correction), whereas the differences disappeared in the Vua condition (100–200 ms: *F*(2,52) = 1.861, *p* = 0.166; 200–300 ms: *F*(2,52)= 1.771, *p* = 0.180).

**Figure 6:**
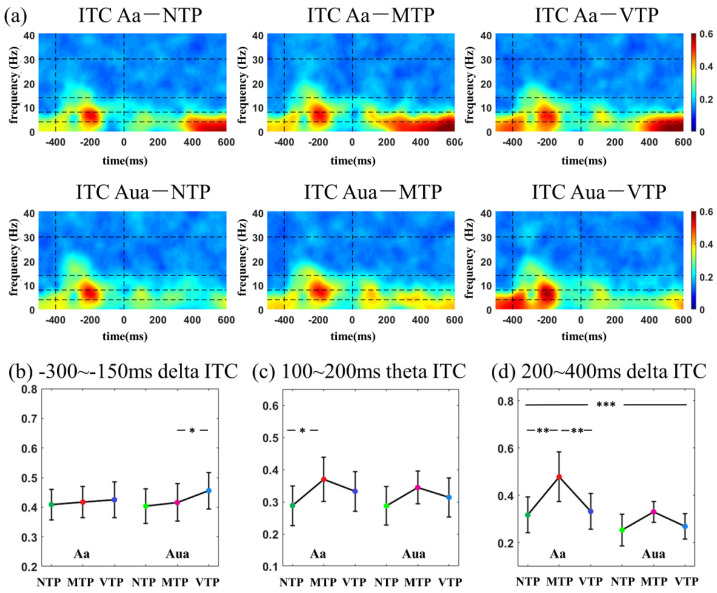
(**a**) ITC time–frequency distributions in auditory modality; comparisons among distinct MA-TP conditions of (**b**)−300–−150 ms delta ITC; (**c**) 100–200 ms theta ITC; (**d**) 200–400 ms delta ITC. Statistical significance: * 0.01 < *p* < 0.05; ** 0.001 < *p* < 0.01; *** *p* < 0.001.

Auditory ITC time-frequency distributions in the six MA-TP conditions were analyzed. The first beep increased delta ITC in −300 to −150 ms temporal window. The second beep increased theta ITC in 100–200 ms delta and 200–400 ms (Figure 6a). The delta ITCs induced by the first beep were almost the same across six conditions. There was no interactive effect (*F*(2,52) = 1.551, *p* = 0.222), MA main effect (*F*(1,26) = 0.124, *p* = 0.728), or TP main effect (*F*(2,52) = 2.857, *p* = 0.052). After the second beep, 100–200 ms theta ITC had a TP main effect (*F*(2,52) = 7.072, *p* = 0.002, $\eta_{p}^{2}$ = 0.214), in which MTP resulted in the largest ITC (NTP vs. MTP: *p* < 0.05; MTP vs. VTP: *p* = 0.289, after Bonferroni correction). In 200–400 ms temporal window, delta ITC was increased in Aa conditions compared with Aua (*F*(1,26) = 11.185, *p* = 0.003, $\eta_{p}^{2}$ = 0.309). There was also a TP main effect (*F*(2,52) = 14.265, *p* < 0.001, $\eta_{p}^{2}$ = 0.363). In Aa condition, MTP had the largest ITC (*F*(2,52) = 12.730, *p* < 0.001, MTP vs. NTP: *p* = 0.001; MTP vs. VTP: *p* = 0.002). No TP-related difference was found in Aua condition.

Within 400 ms after the second stimulus onset, TP caused theta ITC variations in Va condition, and delta variations in Aa condition. However, the differences induced by the TP disappeared in both the Vua and Aua condition. This suggests that the neural effect of TP only evident when the MA was allocated to the corresponding modality.

### 3.4. ERSP Time–Frequency Analyses

**Figure 7:**
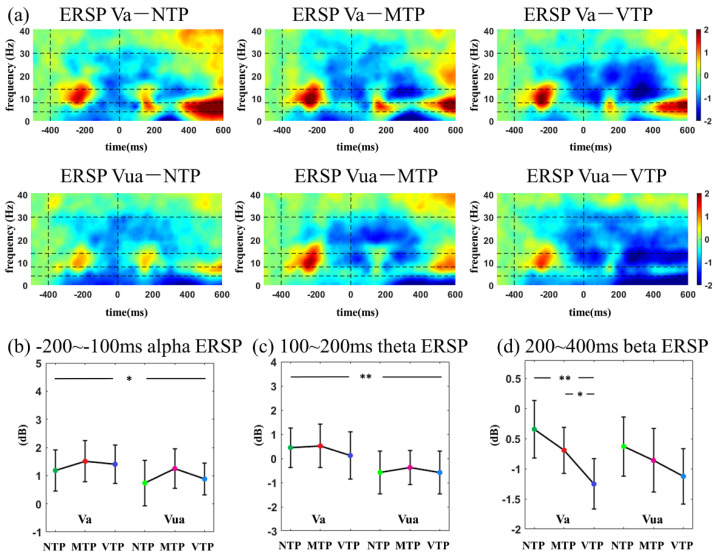
(**a**) ERSP time–frequency distributions in visual modality; comparisons among distinct MA-TP conditions of (**b**)−200–−100 ms alpha ERSP; (**c**) 100–200 ms theta ERSP; (**d**) 200–400 ms beta ERSP. Statistical significance: * 0.01 < *p* < 0.05; ** 0.001 < *p* < 0.01.

ERSP time-frequency distributions in the visual modality can be found in Figure 7. In the −200 to −100 ms period, Va conditions had a larger alpha ERSP than Vua (*F*(1,26) = 5.937, *p* = 0.022, $\eta_{p}^{2}$ = 0.186). There was no TP main effect (*F*(2,52) = 2.252, *p* = 0.059) or interactive effect (F(2,52) = 0.376, *p* = 0.688). After the second flash, 100–200 ms theta ERSP had attentional enhancement (100–200 ms: *F*(1,26) = 11.572, *p* = 0.002, $\eta_{p}^{2}$ = 0.308). In the 200–400 ms period under Va conditions, VTP resulted in a smaller beta ERSP than others (*F*(2,52) = 11.007, *p* < 0.001, NTP vs. VTP: *p* < 0.01, MTP vs. NTP: *p* = 0.016, after Bonferroni correction).

**Figure 8:**
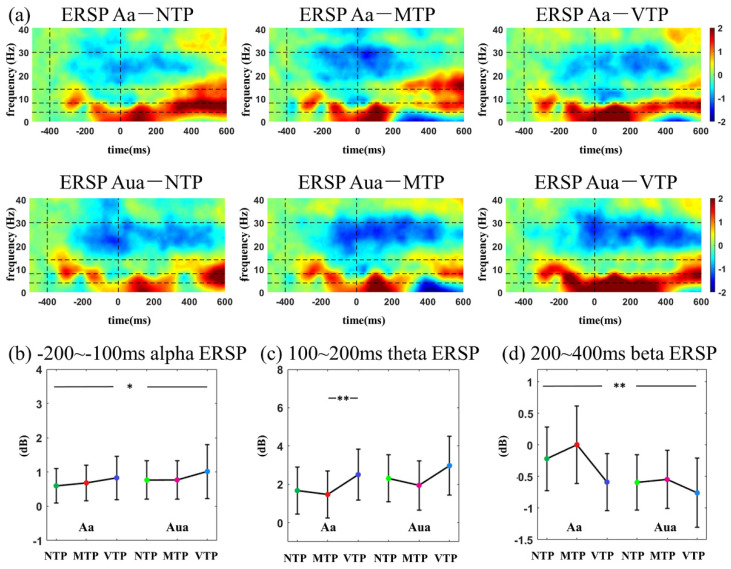
(**a**) ERSP time−frequency distributions in auditory modality; comparisons among distinct MA-TP conditions of (**b**)−200–−100 ms alpha ERSP; (**c**) 100–200 ms theta ERSP; (**d**) 200–400 ms beta ERSP. Statistical significance: * 0.01 < *p* < 0.05; ** 0.001 < *p* < 0.01.

ERSP time-frequency distributions in auditory modality can be found in Figure 8. In the −200 to −100 ms period, auditory attention enhanced alpha ERSP (*F*(1,26) = 7.059, *p* = 0.013, $\eta_{p}^{2}$ = 0.214). After the second beep, an increased ERSP first emerged, but showed no difference among conditions. Then, there was a beta suppression, which revealed a MA main effect (*F*(1,26) = 10.813, *p* = 0.003, $\eta_{p}^{2}$ = 0.294).

MA led to larger alpha ERSPs in both the visual and auditory modalities after the first stimulus onset. After the second stimulus, attention increased theta ERSP. Beta differences induced by the TP were only found in Va condition.

## 4. Discussion

This study investigated how the MA and precise single-interval TP modulated early sensory responses. We found in the visual modality, after the predicted moment (for example, the second flash), distinct TP conditions affected N1-N2 amplitude. Theta ITC differences were only observed in the Va condition, no difference was seen in Vua conditions. In the auditory modality, distinct TP conditions led to P2 amplitude variations and delta ITC differences only in the Aa condition, no difference was found in the Aua condition. These results suggest that the MA increased the TP-related response differences.

### 4.1. The MA-TP Mainly Modulated Low-Frequency ITCs in Early Sensory Processing

We first analyzed ERP signatures, including the N1-N2 component in visual modality, P2 component in auditory modality. The differences induced by distinct MA-TP conditions were small (which was measured by the parameter $\eta_{p}^{2}$). A possible reason for this may be that the MA-TP changes were hidden by the low-frequency ERPs, which had very large amplitudes. Therefore, it is necessary to analyze neural activities in distinct frequency bands.

As expected, ITC time-frequency distributions had more obvious distinctions amongst the six MA-TP conditions in both the visual and auditory modalities. In the visual modality, alpha ITC was significantly increased after the first flash by the MA, but was not sensitive to TP modulation. Furthermore, the theta ITC was sensitive to both MA and TP modulations and there was a significant MA-TP interaction. Theta-band activity is traditionally related to specific cognitive controls [33], such as maintenance of working memory [34]; sustained attention [35]; shift of spatial attention [36]; and prediction errors management in perceptual learning [15,37]. A recent study reported that theta ITC is instrumental in shaping temporal predictions in early sensory processing [23]. For example, in rhythmic predictive timing process, phase-reset aligns stimulus and the ideal phase of delta-theta oscillation, which is correlated with following evoked ERPs [23]. In a time estimation task with rotating intervals, theta ITC in the frontal area was modulated by error magnitude, possibly indexing the degree of surprise [15]. The current study found that the theta ITC not only had a close association with the TP, but also reflected the interaction between the MA and TP, which went beyond the traditional role of theta ITC. Furthermore, previous studies proposed that the posterior theta is related to stimulus processing, but unaffected by task demands [31]. Although this study found specific theta changes could reflect top-down modulations in the posterior brain (O1, Oz, O2). Such observations suggest that the top-down modulation to theta activity can be observed not only in frontal area, but also in the primary visual cortex.

The auditory ITC is primarily located in delta and theta bands, of which the differences were in lower frequency bands than the visual responses. The delta phase synchronization has been widely accepted as a neural mechanism underlying rhythmic timing prediction [23], recent studies further demonstrated that delta phase works as neural mechanism of single-interval timing prediction as well [14]. Therefore, in the current single-interval precise TP cognitive process, it is reasonable to observe delta ITC changes among distinct TP conditions. ITC in the 200–400 ms period was concurrently modulated by the MA and TP. To the best of our knowledge, this may be novel neural evidence regarding the effect of delta ITC.

Additionally, the alpha and beta ERSP were affected by MA or TP. After the first stimulus onset, smaller alpha ERSP was found in both the Va and Aa conditions. Alpha oscillation has a key role in many mental tasks [38], especially the attention process [17,39]. Therefore, it is reasonable to observe MA-related alpha changes here. The beta ERSP was modulated by the TP in Va conditions. Many predictive timing studies have reported the suppression in post-stimulus beta power, and data suggest that it may have a key role in time maintenance or prediction error encoding [40,41,42,43]. This study found much smaller beta ERSP 200–400 ms after the second flash in Va conditions. This suggests that VTP may have a longer period for time maintenance or have larger prediction error encodings.

### 4.2. Visual MA Promoted the TP-Related Neural Effect in N1 and N2 Period

In visual modality, the NTP, MTP and VTP responses of single-interval precise TP were compared in Va and Vua conditions. In the 100–300 ms (N1) period of the Va condition, MTP had almost the same ERP and ITC performance as the NTP, which was the least influenced by top-down factors. VTP had a much smaller theta ITC than the MTP. In the 300–400 ms (N2) period, the VTP led to much larger negative waveforms than others. Allowing for the TP is a neural implementation of the predictive coding in time domain [23,24], N1 and N2 performance may be explained by the ‘sharpen’ and ‘dampen’ effects of predictive coding theory, respectively. According to the predictive coding, there are two types of neurons in a prediction process. One type of neuron encodes the information that is the same as the expected feature, the other type of neuron encodes the information that is different from expected (i.e., prediction error). The ‘sharpen’ effect proposed that the neurons which are not tuned to the expected information that is suppressed, making the expected features more salient and selective. The ‘dampen’ effect suggests that the information which is different from expected feature would result in larger responses, which was used for encoding prediction errors [44,45,46]. Correspondingly, the VTP condition, which represented unexpected information, was suppressed, whereas the MTP condition was not influenced, as it had almost the same ITC as the NTP. Such observations were consistent with the ‘sharpen’ effect. Regarding the N2 variations, VTP resulted in a more negative waveform than the MTP in Va conditions. Negative waveforms have been suggested as a neural representation of prediction error encodings [47,48], and the ‘dampen’ effect may explain this negative waveform. Therefore, in Va conditions, we found ‘sharpened’ N1 and ‘dampened’ N2 performance.

These results demonstrate that when MA existed in the visual modality, N1 was sharpened and N2 was dampened. This supports the results of our previous study. This was observed even when the brain was concurrently faced with visual-auditory stimuli. However, such N1-N2 performance disappeared when the MA was not attended in the visual modality, which suggests that the MA promoted the neural effects of the TP in the visual modality.

### 4.3. Auditory MA Also Promoted the TP-Related Neural Effect

We then investigated auditory neural responses. In Aa condition, the P2 component different in the VTP and MTP conditions. MTP went rapidly trended up 100 ms after the second beep. VTP had a more gradual upward trend, leading to a relatively negative waveform. This negative waveform may also be explained by the ‘dampen’ effect, which reflects the encodings of prediction error. However, in Aua condition, no significant difference was found, suggesting that the auditory MA promoted the TP neural effect.

There were some differences between the TP neural effect in visual and auditory modality. Compared with the visual results, auditory responses had clear TP-related differences in the lower frequency band. Visual responses revealed both the sharpen and dampen effects, but the auditory P2 only showed only the dampen effect. There are two potential explanations for this phenomenon. Early sensory processing may be essentially different when the brain is faced with visual and auditory responses. Alternatively, auditory ERP is primarily located in the frontal-central area, which is also the area neural circuits of predictive timing are found. This could mean that the neural signatures reflecting early auditory processing may be confused with the signals related to the top-down controls for higher cognitive processing. Therefore, it is necessary to investigate how to separate purely auditory response from the prediction-related variations in frontal area, if we want to have a better understanding of the neural effect of TP in auditory modality.

## 5. Conclusions

This study investigated how visual-auditory modality attention influences the neural effect of single-interval precise TP. We found in both the visual and auditory modality, the MA increased TP-related differences. Sharpened N1 and dampened N2 were only found in Va conditions, whereas dampened P2 was only found in Aa conditions. These results may provide new neural evidence for our understanding of the interactions between the MA and TP.

## Data Availability

Please contact the email mengjiayuan@tju.edu.cn to get the access of data.

## References

[1] Buhusi C.V., Meck W. (2005). What makes us tick? Functional and neural mechanisms of interval timing. Nat. Rev. Neurosci..

[2] Nobre A.C., van Ede F. (2018). Anticipated moments: Temporal structure in attention. Nat. Rev. Neurosci..

[3] Breska A., Ivry R.B. (2018). Double dissociation of single-interval and rhythmic temporal prediction in cerebellar degeneration and Parkinson’s disease. Proc. Natl. Acad. Sci. USA.

[4] Shalev N., Nobre A.C., van Ede F. (2019). Time for What? Breaking Down Temporal Anticipation. Trends Neurosci..

[5] Bueti D., Lasaponara S., Cercignani M., Macaluso E. (2012). Learning about Time: Plastic Changes and Interindividual Brain Differences. Neuron.

[6] Coull J.T., Droit-Volet S. (2018). Explicit Understanding of Duration Develops Implicitly through Action. Trends Cogn. Sci..

[7] Morillon B., Baillet S. (2017). Motor origin of temporal predictions in auditory attention. Proc. Natl. Acad. Sci. USA.

[8] Heys J.G., Wu Z., Mascaro A.L.A., Dombeck D.A. (2020). Inactivation of the Medial Entorhinal Cortex Selectively Disrupts Learning of Interval Timing. Cell Rep..

[9] Spence C., Driver J. (1997). On measuring selective attention to an expected sensory modality. Percept. Psychophys..

[10] Pfeuty M., Ragot R., Pouthas V. (2005). Relationship between CNV and timing of an upcoming event. Neurosci. Lett..

[11] Mento G., Tarantino V., Sarlo M., Bisiacchi P.S. (2013). Automatic Temporal Expectancy: A High-Density Event-Related Potential Study. PLoS ONE.

[12] van Rijn H., Kononowicz T.W., Meck W.H., Ng K.K., Penney T.B. (2011). Contingent negative variation and its relation to time estimation: A theoretical evaluation. Front. Integr. Neurosci..

[13] Stefanics G., Hangya B., Hernádi I., Winkler I., Lakatos P., Ulbert I. (2010). Phase entrainment of human delta oscillations can mediate the effects of expectation on reaction speed. J. Neurosci..

[14] Daume J., Wang P., Maye A., Zhang D., Engel A.K. (2020). Non-rhythmic temporal prediction involves phase resets of low-frequency delta oscillations. Neuroimage.

[15] Barne L.C., Claessens P.M.E., Reyes M.B., Caetano M.S., Cravo A.M. (2017). Low-frequency cortical oscillations are modulated by temporal prediction and temporal error coding. Neuroimage.

[16] Solís-Vivanco R., Jensen O., Bonnefond M. (2018). Top–Down Control of Alpha Phase Adjustment in Anticipation of Temporally Predictable Visual Stimuli. J. Cogn. Neurosci..

[17] Samaha J., Bauer P., Cimaroli S., Postle B.R. (2015). Top-down control of the phase of alpha-band oscillations as a mechanism for temporal prediction. Proc. Natl. Acad. Sci. USA.

[18] van Ede F., de Lange F., Jensen O., Maris E. (2011). Orienting attention to an upcoming tactile event involves a spatially and temporally specific modulation of sensorimotor alpha- and beta-band oscillations. J. Neurosci..

[19] Chen F.-W., Li C.-H., Kuo B.-C. (2023). Temporal expectation based on the duration variability modulates alpha oscillations during working memory retention. Neuroimage.

[20] Rao R.P.N., Ballard D.H. (1999). Predictive coding in the visual cortex: A functional interpretation of some extra-classical receptive-field effects. Nat. Neurosci..

[21] Friston K. (2005). A theory of cortical responses. Philos. Trans. R Soc. Lond. B Biol. Sci..

[22] De Lange F.P., Heilbron M., Kok P. (2018). How do expectations shape perception?. Trends Cogn. Sci..

[23] Arnal L.H., Giraud A.-L. (2012). Cortical oscillations and sensory predictions. Trends Cogn. Sci..

[24] Sherwell C., Garrido M.I., Cunnington R. (2017). Timing in Predictive Coding: The Roles of Task Relevance and Global Probability. J. Cogn. Neurosci..

[25] Kononowicz T.W., van Rijn H. (2014). Decoupling interval timing and climbing neural activity: A dissociation between CNV and N1P2 amplitudes. J. Neurosci..

[26] Xu M., Meng J., Yu H., Jung T.-P., Ming D. (2020). Dynamic Brain Responses Modulated by Precise Timing Prediction in an Opposing Process. Neurosci. Bull..

[27] Plass J., Ahn E., Towle V.L., Stacey W.C., Wasade V.S., Tao J., Wu S., Issa N.P., Brang D. (2019). Joint Encoding of Auditory Timing and Location in Visual Cortex. J. Cogn. Neurosci..

[28] Dean C.L., Eggleston B.A., Gibney K.D., Aligbe E., Blackwell M., Kwakye L.D. (2017). Auditory and visual distractors disrupt multisensory temporal acuity in the crossmodal temporal order judgment task. PLoS ONE.

[29] Keil J., Pomper U., Feuerbach N., Senkowski D. (2017). Temporal orienting precedes intersensory attention and has opposing effects on early evoked brain activity. Neuroimage.

[30] Delorme A., Makeig S. (2004). EEGLAB: An Open Source Toolbox for Analysis of Single-Trial EEG Dynamics Including Independent Component Analysis. J. Neurosci. Methods.

[31] Bidelman G.M., Myers M.H. (2019). Frontal cortex selectively overrides auditory processing to bias perception for looming sonic motion. Brain Res..

[32] Sadeghijam M., Moossavi A., Akbari M., Yousefi A., Haghani H. (2022). Effect of tinnitus distress on auditory steady-state response am-plitudes in chronic tinnitus sufferers. J. Clin. Neurosci..

[33] Caravaglios G., Muscoso E.G., Di Maria G., Costanzo E. (2012). Theta responses are abnormal in mild cognitive impairment: Evidence from analysis of theta event-related synchronization during a temporal expectancy task. J. Neural Transm..

[34] Gevins A., Smith M.E. (2000). Neurophysiological Measures of Working Memory and Individual Differences in Cognitive Ability and Cognitive Style. Cereb. Cortex.

[35] Chang C.-F., Liang W.-K., Lai C.-L., Hung D.L., Juan C.-H. (2016). Theta Oscillation Reveals the Temporal Involvement of Different Attentional Networks in Contingent Reorienting. Front. Hum. Neurosci..

[36] Schrooten M., Ghumare E.G., Seynaeve L., Theys T., Dupont P., Van Paesschen W., Vandenberghe R. (2017). Electrocorticography of spatial shifting and attentional selection in human superior parietal cortex. Front. Hum. Neurosci..

[37] Lundin N.B., Bartolomeo L.A., O’Donnell B.F., Hetrick W.P. (2018). Reduced electroencephalogram responses to standard and target auditory Stimuli in bipolar disorder and the impact of psychotic features: Analysis of event-related potentials, spectral power, and inter-trial coherence. Bipolar Disord..

[38] Jensen O., Mazaheri A. (2010). Shaping Functional Architecture by Oscillatory Alpha Activity: Gating by Inhibition. Front. Hum. Neurosci..

[39] Deiber M.-P., Meziane H.B., Hasler R., Rodriguez C., Toma S., Ackermann M., Herrmann F., Giannakopoulos P. (2015). Attention and Working Memory-Related EEG Markers of Subtle Cognitive Deterioration in Healthy Elderly Individuals. J. Alzheimer Dis..

[40] Meijer D., Woerd E.T., Praamstra P. (2016). Timing of beta oscillatory synchronization and temporal prediction of upcoming stimuli. Neuroimage.

[41] Arnal L.H., Doelling K.B., Poeppel D. (2015). Delta–Beta Coupled Oscillations Underlie Temporal Prediction Accuracy. Cereb. Cortex.

[42] Andersen L.M., Dalal S.S. (2021). The cerebellar clock: Predicting and timing somatosensory touch. Neuroimage.

[43] Betti V., Della Penna S., de Pasquale F., Corbetta M. (2020). Spontaneous Beta Band Rhythms in the Predictive Coding of Natural Stimuli. Neurosci..

[44] Kok P., Jehee J., de Lange F. (2012). Less Is More: Expectation Sharpens Representations in the Primary Visual Cortex. Neuron.

[45] Yon D., Gilbert S.J., de Lange F.P., Press C. (2018). Action sharpens sensory representations of expected outcomes. Nat. Commun..

[46] Press C., Kok P., Yon D. (2019). The Perceptual Prediction Paradox. Trends Cogn. Sci..

[47] Folstein J.R., Van Petten C. (2008). Influence of cognitive control and mismatch on the N2 component of the ERP: A review. Psychophysiology.

[48] Luck S.J., Kappenman E.S. (2012). The Oxford Handbook of Event-Related Potential.

